# Fraternal twins: Swiprosin-1/EFhd2 and Swiprosin-2/EFhd1, two homologous EF-hand containing calcium binding adaptor proteins with distinct functions

**DOI:** 10.1186/1478-811X-9-2

**Published:** 2011-01-18

**Authors:** Sebastian Dütting, Sebastian Brachs, Dirk Mielenz

**Affiliations:** 1 Division of Molecular Immunology, Department of Medicine III, Nikolaus Fiebiger Center, University of Erlangen-Nürnberg, 91054 Erlangen, Germany

## Abstract

Changes in the intracellular calcium concentration govern cytoskeletal rearrangement, mitosis, apoptosis, transcriptional regulation or synaptic transmission, thereby, regulating cellular effector and organ functions. Calcium binding proteins respond to changes in the intracellular calcium concentration with structural changes, triggering enzymatic activation and association with downstream proteins. One type of calcium binding proteins are EF-hand super family proteins. Here, we describe two recently discovered homologous EF-hand containing adaptor proteins, Swiprosin-1/EF-hand domain containing 2 (EFhd2) and Swiprosin-2/EF-hand domain containing 1 (EFhd1), which are related to allograft inflammatory factor-1 (AIF-1). For reasons of simplicity and concision we propose to name Swiprosin-1/EFhd2 and Swiprosin-2/EFhd1 from now on EFhd2 and EFhd1, according to their respective gene symbols. AIF-1 and Swiprosin-1/EFhd2 are already present in *Bilateria*, for instance in *Drosophila melanogaster *and *Caenhorhabditis elegans*. Swiprosin-2/EFhd1 arose later from gene duplication in the tetrapodal lineage. Secondary structure prediction of AIF-1 reveals disordered regions and one functional EF-hand. Swiprosin-1/EFhd2 and Swiprosin-2/EFhd1 exhibit a disordered region at the N-terminus, followed by two EF-hands and a coiled-coil domain. Whereas both proteins are similar in their predicted overall structure they differ in a non-homologous stretch of 60 amino acids just in front of the EF-hands. AIF-1 controls calcium-dependent cytoskeletal rearrangement in innate immune cells by means of its functional EF-hand. We propose that Swiprosin-1/EFhd2 as well is a cytoskeleton associated adaptor protein involved in immune and brain cell function. Pro-inflammatory conditions are likely to modulate expression and function of Swiprosin-1/EFhd2. Swiprosin-2/EFhd1, on the other hand, modulates apoptosis and differentiation of neuronal and muscle precursor cells, probably through an association with mitochondria. We suggest furthermore that Swiprosin-2/EFhd1 is part of a cellular response to oxidative stress, which could explain its pro-survival activity in neuronal, muscle and perhaps some malignant tissues.

## Introduction

Calcium ions (Ca^2+^) regulate enzyme activities, cytoskeletal and transcriptional regulation, mitosis or apoptosis, gene expression, synaptic communication and homeostasis of reactive oxygen species (ROS) [1]. Changes in the intracellular Ca^2+ ^concentration therefore control cellular effector as well as organ functions, such as immune cell activation or brain function, to name only a few. Ca^2+ ^signaling is a consequence of temporary and/or local increases in the intracellular Ca^2+ ^concentration, through the opening of Ca^2+ ^channels in the endoplasmic reticulum (ER) and the plasma membrane. Ca^2+ ^acts as a ubiquitous, diffusible second messenger that is stored in the ER and mitochondria. It diffuses from the ER into the cytoplasm due to opening of Ca^2+ ^channels in the ER membrane in response to increases in inositol-1,4,5-trisphosphate (IP_3_), a metabolite of the cleavage of phosphatidylinositol-4,5-bisphosphate (PIP_2_) by members of the PI-phospholipase C family of enzymes. A wide variety of cell surface receptors including growth factor receptors, cytokine receptors, G-protein coupled receptors (GPCR), integrins, the high affinity receptor for immunoglobulin E (IgE) - FcεR1 - on mast cells as well as B and T cell receptors (BCR and TCR) can activate phospholipase C enzymes directly and indirectly through different mechanisms [2]. The depletion of intracellular Ca^2+ ^stores and the concomitant rise in the cytosolic Ca^2+ ^concentration induces influx of extracellular Ca^2+ ^across the plasma membrane through store operated calcium entry (SOCE) and calcium release activated calcium (CRAC) channels. Key players in this process are stromal interaction molecules 1 and 2 (STIM1 and 2), which sense the depletion of ER Ca^2+ ^stores, and ORAI1, which represents a pore subunit of the CRAC channel (for review see references [3,4]).

Ca^2+^-binding proteins of the EF-hand super family [5-7] are involved in all of the above-mentioned cellular processes. EF-hand bearing proteins are heterogeneous in their structures and calcium binding properties. R. H. Kretsinger, who discovered a Ca^2+^-binding helix-loop-helix motif in the structure of Parvalbumin [8], named this motif "EF-hand". The corresponding linear sequence motif (Pfam PF00036; http://pfam.sanger.ac.uk/) has thereafter been discovered in the amino acid sequences of many other calcium-binding proteins [9-13]. Here, we provide information about the recently described EF-hand proteins Swiprosin-1/EFhd2 and Swiprosin-2/EFhd1, which are related to another EF-hand protein, allograft inflammatory factor-1 (AIF-1; also: Iba1, ionized calcium binding adapter molecule 1). Although our own work focuses on B cell development and signaling we provide information for a broader readership here. There are many synonyms for Swiprosin/EFhd genes, transcripts and proteins (Additional file 1, Table S1). Whereas the nomenclature of the genes is unambiguous, alternative names complicate work with the proteins (Additional file 1, Table S1). For simplicity and concision, we propose to name the proteins from now on EFhd1 and EFhd2, according to their respective gene symbols. Hence, we will refer in this review and future publications to Swiprosin-1/EFhd2 as EFhd2 and to Swiprosin-2/EFhd1 as EFhd1.

**Figure 1:**
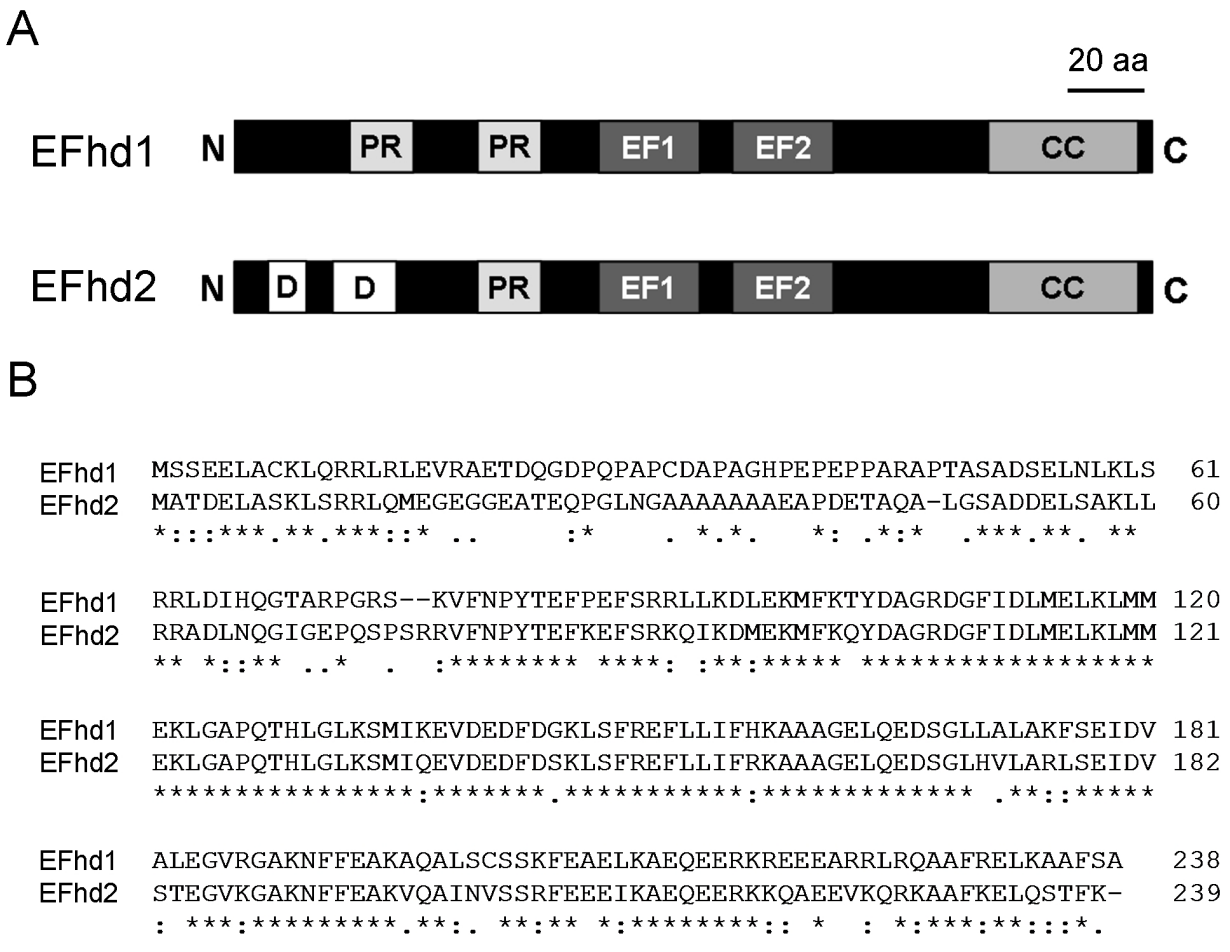
**Predicted secondary structures and sequence comparisons of EFhd2 and EFhd1**. **a) Predicted secondary structures of murine EFhd1 and EFhd2 (240 aa) (http://elm.eu.org). **PR: proline-rich elements (potential SH3 domain-binding sites), EF: EF-hands, CC: coiled-coil domain, D: disordered region. **b) Aligment of murine EFhd1 (Q9D4J1) and EFhd2 (Q9D8Y0) sequences using ClustalW2 (http://www.ebi.ac.uk/clustalw)**. Amino acid (aa) positions are marked on the right. "*", identical aa, ":", conserved aa, "." semi conserved aa.

EFhd2 and EFhd1 exhibit similar predicted secondary structures (http://elm.eu.org) (Figure 1a), with disordered regions at the N-terminus, predicted and functional SH3-domain binding sites [14], followed by two EF-hands and a coiled-coil domain at the C-terminus. Both proteins bind indeed Ca^2+ ^[15,16] and exhibit a high degree of sequence identity at the protein level (64.58%) (Figure 1b). The only significantly different region are amino acids (aa) 20-80 (referring to the numbering of EFhd2). A part of this region (aa 70-78) has been shown by us to be important for targeting EFhd2 to detergent resistant membranes (DRM) in the murine B cell line WEHI231 [14]. Both EFhd2 [14] and EFhd1 (Additional file 2, Figure S1) are conserved amongst species, with an orthologue of EFhd2 in *Drosophila melanogaster *(CG10641). Interestingly, regarding EFhd2 and EFhd1 from the same species (Figure 1a), or either EFhd2 [14] and EFhd1 (Additional file 2, Figure S1) from different species, aa 20-80 differ the most. Since EFhd2 and EFhd1 are so similar in their other domains, this part of the proteins is likely to specify distinct functions of EFhd2 and EFhd1. One would therefore assume that EFhd2 and EFhd1 bind proteins redundantly as well as uniquely. When expressed in the same cellular context, EFhd2 and EFhd1 might transmit Ca^2+ ^signals into a different signaling outcome, especially because there is so far no evidence for an interaction of EFhd2 with EFhd1 (D.M. et al., unpublished data).

## Evolution of the genes encoding AIF-1 (*aif1*), EFhd2 (*efhd2*) and EFhd1 (*efhd1*)

**Figure 2:**
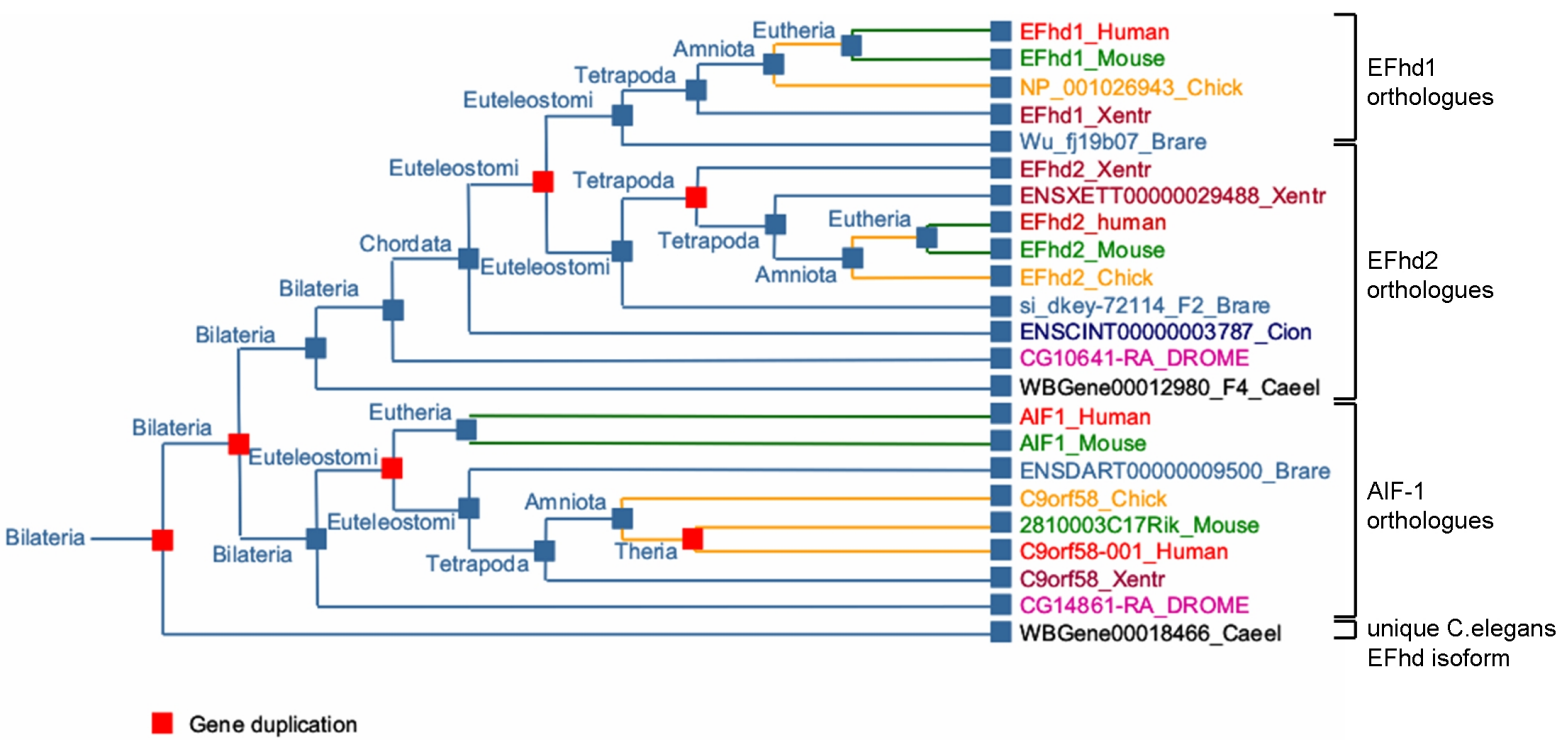
**Phylogeny of EFhd genes**. A phylogenetic tree with equal distances between different taxons was generated using *efhd1 *as search parameter (http://www.treefam.org/cgi-bin). Search parameters were: accession number TF320736 / clean / equal distance / show taxa / none / exp / selected species. Only representative species are shown for convenience. On the right hand side the employed transcript sequences are shown. Chick, chicken (*Gallus gallus*), Brare, zebrafish (*Danio rerio*), Xentr, frog (*Xenopus tropicalis*), Cioin, Hydra (*Ciona intestinalis*), Drome, fruitfly (*Drosophila melanogaster*), Caeel, worm (*Caenorhabtidis elegans*).

The first *efhd/aif1 *precursor gene is present in an ancestral species of the animal taxon *Bilateria *(Figure 2; http://www.treefam.org/cgi-bin). This ancient gene underwent duplication in an unknown species, giving rise to two branches. One branch represents *efhd *and *aif1*, both of which gene products exhibit EF-hand domains (one in AIF-1 and two in EFhd). The other branch represents a gene encoding a unique protein in *C. elegans *(WP:CE38519) (Figure 2, bottom) that consists only of a coiled-coil domain at the C-terminus (not shown). The *efhd *gene products contain a disordered region, two central EF-hands and a C-terminal coiled-coil domain whereas AIF-1 contains only a disordered region and two EF-hands, the second of which is degenerate. Accordingly, the homology between *aif1 *and *efhd *genes is restricted to the region encoding the EF-hand domains (representative result for murine EFhd2 is shown in Additional file 3, Figure S2). This suggests that calcium binding is the most prominent feature of this protein family. The *aif1/efhd *precursor underwent a second duplication, resulting in *aif1 *genes and the *efhd2 *precursor. The *efhd2 *gene is already present in insects where it represents the only *efhd *gene (Figure 2). In one species of the taxon *Euteleostomi*, after the onset of *Chordata *development, the ancient *efhd2 *gene was duplicated once more, now representing two genes, *efhd2 *and *efhd1*, encoding for the proteins EFhd2 and EFhd1. These are - amongst other species - present in man, mouse, rat, chicken, zebrafish and frog. In the tetrapodal lineage, putatively during or before development of *Xenopus spec*., there was yet another duplication of the *efhd2 *gene. Thus, *Xenopus tropicalis *contains three *efhd *genes, *efhd1*, *efhd2 *and a third unique gene that is still lacking a proper name (*ensxett00000029488_xentr*). Interestingly, the gene product of *ensxett00000029488_xentr *contains no EF-hands, but only disordered regions and a coiled-coil domain. This protein might therefore compete with the EF-hand containing *Xenopus *EFhd2 and EFhd1 for calcium-independent binding partners. In summary, EFhd2 is the ancestral protein, being present for instance in worms and insects, and EFhd2 and EFhd1 co-exist from the euteleostomic lineage on, for instance in zebrafish, frog and later evolutionary stages. This provokes the question as to whether the gene duplication resulting in the *efhd1 *gene provided an evolutionary advantage.

## Chromosomal location of the human and murine genes *efhd2 *and *efhd1*

The murine *efhd2 *gene is located on chromosome 4 (see http://www.ensembl.org for further details). It contains 4 exons with a long intron between exon 1 and 2 (15 kb) and a long 3'-UTR in exon 4 (approx. 1.6 kb). This structure is conserved in humans where the *efhd2 *gene is located on chromosome 1. The murine *efhd2 *gene is located centrally in a susceptibility locus for SLE (Systemic Lupus Erythematodes) (LMB-1, close to D4Mit33) on chromosome 4 [17]. The human *efhd2 *gene lies in the PARK7 locus (1p36.33 - 1p36.12) that is associated with autosomal recessive early onset Parkinson's disease [18]. For the human *efhd2 *gene product, EFhd2, three protein-coding splice variants have been predicted, translating into putative protein products of 177 to 240 aa. The murine *efhd1 *gene is localized on chromosome 1. It is also comprised of 4 exons with an even longer intron between exons 1 and 2 (approx. 25 kb), similar to human *efhd1 *(chromosome 2). For the human *efhd1 *gene product, EFhd1, five protein-coding splice variants are predicted, resulting in potential protein products of 81 to 239 aa.

## EFhd2

### Expression of EFhd2

An enhancer trap screen has identified a *Drosophila *strain with an intriguing expression pattern in muscle cells and somatic muscle precursor cells. The insertion is in the *Drosophila efhd *homologue, CG10641 [19]. Furlong et al. [20] revealed expression of CG10641 by microarray analyses and in situ hybridization during mesoderm development. Estrada et al. show expression of CG10641 in the visceral and somatic mesoderm, stage 9 ventral head mesoderm, stage 10 head mesoderm and in hemocytes but did not follow up the expression patter of CG10641 during mesoderm differentiation [21]. However, based on these expression data Kiefer et al. [22] propose a function for all *Drosophila *CG10641-related genes in muscle. Indeed, Renate Renkawitz-Pohl's group (C. Hornbruch and R. Renkawitz-Pohl, personal communication) shows expression of *Drosophila *EFhd (CG10641 encoded) in hematocytes and during myoblast fusion. The EFhd2 orthologue in *C. elegans *is expressed in pharynx, body wall muscle, the nervous system and the ventral nerve cord. In zebrafish the associated ESTs are expressed in brain, eye, genitourinary tissue, gills, muscle, and olfactory rosettes. In *C. elegans*, zebrafish and mouse the respective orthologues may have additional functions, including a role in the nervous system [22]. The latter appears to be true in human and mouse, at least under pathological conditions such as neurodegeneration, schizophrenia and alcohol addiction [15,23-25].

Muscle is derived from mesoderm but the mesoderm gives also rise for instance to bone (osteoblasts and osteoclasts), connective tissue and hematopoietic cells. In humans, EFhd2 becomes transiently up-regulated during osteoblast differentiation from human mesenchymal stem cells [26]. EFhd2 is also up-regulated during differentiation of the murine RAW264 macrophage cell line into osteoclasts after treatment with RANK-L (receptor activator of NF-κB ligand) [27]. Other cell types of mesodermal origin expressing EFhd2 are particularly immune cells: EFhd2 is expressed in human and primary murine mast cells [[28,29]; our unpublished data], human and murine B cells [30-32], in CD4^+ ^and CD8^+ ^T cells [32-34], natural killer cells (NK cells) [35] and human peripheral blood mononuclear cells (PBMC) [36,37]. PBMC are a heterogeneous cell population of lymphocytes (~75% CD4^+ ^and CD8^+ ^T cells and ~25% NK and B cells), monocytes and macrophages. In accordance, murine *efhd2 *mRNA is present at relatively high abundance in total spleen by Northern Blot, at similar levels in liver and lung, and at relatively low amounts in kidney [31]. The highest expression of *efhd2 *has, however, been detected in ectodermal tissue, namely brain [31]. Likewise, the murine EFhd2 protein was identified in mouse brain [38], specifically in brain stem, cerebellum, amygdala, striatum, cortex and frontal lobe [15] as well as in the hippocampus [15,23]. With the exception of microglia cells [39] it remains to be established which cell type(s) in the brain express EFhd2. Other cells of ectodermal origin expressing EFhd2 are primary immortalized human keratinocytes [40]. Finally, during mouse development EFhd2 is detected strongly in ectodermal tissue such as the most rostral foregut and the caudal hindgut at E8.5 [41]. In addition, EFhd2 is also down-regulated in embryos deficient for the transcription factor Foxa2 and is a marker of definitive endoderm [41]. Interestingly, EFhd2 is already present in murine embryonic stem cells (our unpublished data). Cell lines expressing EFhd2 are all murine B cell lines we analyzed (our unpublished data), HeLa cells [42] and NIH3T3 cells (our unpublished data). In summary, in worm, zebrafish and higher vertebrates EFhd2 is not only expressed during mesoderm development but also in endodermal and ectodermal tissues. Whether this is also the case in *D. melanogaster *remains to be determined.

### Involvement of EFhd2 in immune cell activation

Innate immune cells respond to pathogen associated molecular patterns (PAMP) and to danger signals released by necrotic cells. Many signaling elements that are important in adaptive immunity have recently also been shown to control innate immunity [43]. Interestingly, signaling molecules of innate immune cells in higher organisms are shared even in *Drosophila *where they control for instance removal of necrotic tissue [43]. Although insects do not possess an adaptive immune system and rely solely on their innate immune system, they utilize ITAMs (immunoreceptor tyrosine based activation motifs). The ITAM is a tyrosine phosphorylation consensus motif (YxxLx_(7-12)_YxxL) [44] found in many receptors and co-receptors of the adaptive and innate immune system as well as in ERM (ezrin, radixin, moesin) proteins [43]. When phosphorylated on their tyrosine residues by Src kinases or Syk (splenic tyrosine kinase) [45], ITAMs bind the SH2 domains of Syk, relieving Syk from autoinhibition and eventually activating it, resulting in a positive feedback loop [45]. Syk is key to signaling from adaptive as well as many innate immune receptors [43,46]. We have recently shown that murine EFhd2 is a positive regulator of Syk activity in response to BCR stimulation in the murine B cell line WEHI231 [14].

Hemocytes of insects are crucial players of immune surveillance by migrating through the body, phagocyting invading pathogens and secreting antimicrobial peptides [47]. One type of hemocytes are the plasmatocytes which resemble mammalian monocytic and macrophage cells [47]. Differentiated macrophage-like hemocytes of *Drosophila *express the *Drosophila *EFhd protein (C. Hornbruch and R. Renkawitz-Pohl, personal communication). This fits to other reports revealing *Drosophila *EFhd expression in hemocytes [48] and in the *Drosophila *S2 cell line which exhibits phagocytotic activity [47]. Many of the hematopoietic factors have been conserved across taxonomic groups [43]. Likewise, EFhd2 is expressed in the murine monocyte cell line RAW264 [27], in human PBMC [36], in microglia cells [39] and in NK-like cells [35]. EFhd2 becomes up-regulated - together with actin - in response to stimulation of the human monocyte cell line THP-1 with a recombinant *Mycobacterium bovis *strain [49]. This stimulation improved the antigen-presenting capacity of THP-1 cells, the CD8^+ ^immune response and TNF-α production [49]. To sum up, EFhd2 is expressed across species in many cell types of the innate and adaptive immune system. It is tempting to speculate that EFhd2 is involved in Syk activation downstream of other immune receptors than the BCR and plays a role in antigen presentation.

### Connection of EFhd2 to the cytoskeleton

Migration and phagocytotic activity of macrophages require a dynamic cytoskeleton. For instance, the small GTPase Cdc42 is required for cellular polarity, formation of filopodia and Fcγ-receptor mediated phagocytosis [50,51]. Rac is required for cell migration and Fcγ-receptor mediated phagocytosis [50,52,53] and Rho is required to retract the trailing edge of migrating cells and for uptake of apoptotic cell fragments [50,54]. EFhd2 is associated with the cytoskeleton in the human mast cell line HMC-1 and in NK-like cells [29,35]. More specifically, EFhd2 is associated in a caspase-9-containing complex with the cytoskeletal protein ezrin [55]. In the brain of a mouse model (JNPL3 mouse) [56] for neurodegeneration (frontotemporal dementia and parkinsonism linked to chromosome 17; FTDP-17), where neurodegeneration and neuroinflammation are induced through transgenic expression of a mutated human tau gene (htau P301L), EFhd2 was identified in a complex with the transgenic human tau protein [15]. In addition, endogenous EFhd2 and tau associate in brain lysates of humans with Alzheimer's disease and FTDP-17 [15]. Finally, EFhd2 becomes dephosphorylated after epidermal growth factor (EGF) stimulation of HeLa cells with the same kinetics as the actin binding protein gelsolin [42]. Taken together, these data demonstrate that EFhd2 is associated with the cytoskeleton but the exact connection is unknown at present. However, the related AIF-1 is associated with the cytoskeleton as well and involved in macrophage survival, migration and activation [57]. Specifically, it augments macrophage phagocytotic activity [58]. Calcium binding to AIF-1 by means of its functional EF-hand is required for its ability to activate Rac or G-CSF expression in vascular smooth muscle cells [59]. Similarly, EFhd2 also binds calcium directly [15] which may lead to interactions with cytoskeletal regulators to modulate functions of innate immune cells, such as cell migration, phagocytosis and antigen presentation. In fact, in the human mast cell line HMC-1 EFhd2 co-localizes with, and modulates F-actin [29].

### Involvement of EFhd2 in NF-κB regulation

**Figure 3:**
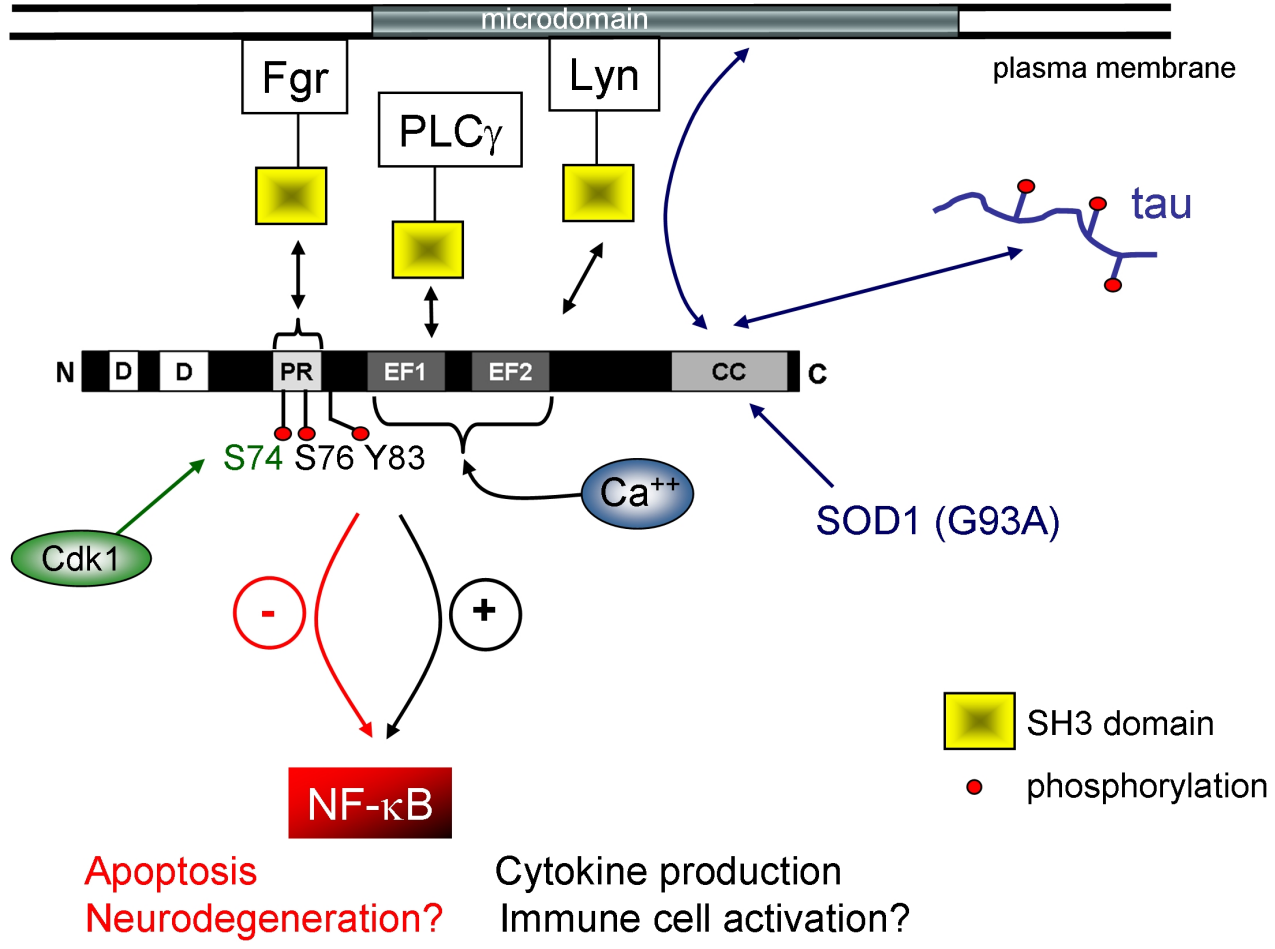
**Involvement of EFhd2 in membrane and cytoskeleton associated pathways**. EFhd2 can associate with detergent resistant membranes (DRM) thought to represent membrane rafts in some B cell lines. Membrane raft association of EFhd2 was also detected in nervous tissue of mice that express a gain of function mutant (G93A) of SOD1. EFhd2 has been shown to exhibit NF-κB-activating as well as NF-κB-inactivating functions in different cell types. Through regulation of NF-κB, EFhd2 may regulate survival or apoptosis of diverse cell types. The interactions of EFhd2 with the SH3 domains of Lyn, PLCγ and Fgr are based on GST-Pull down experiments using recombinant SH3 domains (see Ref. 14). The interactions of Fgr and Lyn SH3 domains with EFhd2 depend on phosphorylation and the SH3 domain of Fgr binds to the predicted proline-rich (PR) SH3 domain binding region of EFhd2. Three phosphorylation sites of EFhd2 are known: S74, S76 and Y83. S74 is a cyclin dependent kinase 1 (Cdk1) site. The ability of EFhd2 to bind calcium may regulate its function and its association with the cytoskeleton. EFhd2 also interacts with hyperphosphorylated Tau in a FTDP17 mouse model as well as in FTDP17 and Alzheimer's patients.

In the human mast cell line HMC-1 (which lacks the Fcε receptor) EFhd2 augments expression of pro-inflammatory cytokines after phorbol myristyl acetate (PMA)/ionomycin stimulation. Similarly, AIF-1 augments expression of IL-6, IL-12 but also IL-10 after lipopolysaccharide (LPS) stimulation of macrophages [60]. Therefore, in macrophages and a mast cell line, perhaps also in primary mast cells (where EFhd2 is expressed; our unpublished data), EFhd2 appears to reveal pro-inflammatory activities, perhaps through positive regulation of the protein kinase C β/nuclear factor κB (PKCβ/NF-κB) signaling pathway [29]. The PKCβ pathway is also activated by the BCR downstream of PLCγ2/Ca^2+ ^signaling and induces eventually NF-κB activation through the Carma1/Bcl10/MALT1 complex [61]. A positive regulation of PKCβ/NF-κB through EFhd2 would therefore be expected as we showed recently that EFhd2 augments BCR-induced Syk/PLCγ2/Ca^2+ ^signaling in the murine B cell line WEHI231 [14]. Unexpectedly, however, we showed already that EFhd2 rather blocks expression of the anti-apoptotic NF-κB target gene *bclxL *[31]. An explanation could be that EFhd2 binds calcium directly [15] and is therefore part of a negative feedback loop preventing NF-κB activation after BCR stimulation (Figure 3). Apoptosis in lymphocytes is effectively controlled by the transcription factor NF-κB [62]. In the murine B cell line WEHI231 that is susceptible to BCR-induced apoptosis, EFhd2 positively regulates spontaneous and BCR-induced apoptosis [31]. Silencing of EFhd2 by shRNA augments survival of WEHI231 cells spontaneously and in response to BCR stimulation. In contrast, G1 cell cycle arrest and p27Kip up-regulation are still induced by BCR ligation in the absence of EFhd2 [31]. To conclude, EFhd2 regulates specifically apoptosis, but not cell cycle arrest in WEHI231 cells. We propose that this is due to suppression of the NF-κB pathway in this cell line. The pro-apoptotic activity of EFhd2 was suppressed by three stimuli that activate NF-κB (CD40 ligation, LPS, and B cell activating factor of the TNF family [BAFF]) [31]. These data seem to oppose data from the mast cell line HMC-1 where EFhd2 augmented up-regulation of NF-kB target genes after PMA/ionomycin stimulation [29], many of which are anti-apoptotic. However, PMA/ionomycin stimulation as used in HMC1 cells [29] and BCR ligation in WEHI231 cells are different stimuli. EFhd2 may enhance BCR-mediated cell death in WEHI231 cells through inhibition of the NF-κB pathway but, through an unknown molecular switch, enhance likewise the NF-κB activating and anti-apoptotic activities of CD40, LPS, and BAFF in combination with BCR stimulation.

### Engagement of EFhd2 in autoimmune disorders

Regulation of the NF-κB pathway by EFhd2, be it positive or negative, together with its expression in innate and adaptive immune cells and its effects on cytokine production [29] argue for involvement of EFhd2 in normal and pathological immune activation. In fact, PBMC of rheumatoid arthritis (RA) patients express less EFhd2 protein than PBMC of healthy patients [36]. Moreover, there is a protease-mediated cleavage of EFhd2 in RA patients [37]. Expression of pro-apoptotic factors in both B and T cells is important to establish tolerance of the adaptive immune system [63]. If EFhd2 was down-regulated in B cells their survival might be prolonged. This might contribute to the persistence of autoimmune diseases such as RA and SLE [64]. Interestingly, the EFhd2-related AIF-1 is involved in RA as well [57]. Moreover, the murine *efhd2 *gene is Iocated rather centrally in an SLE susceptibility region (LMB-1, close to D4Mit33) on chromosome 4 [17], thereby, representing a putative genetic determinant of SLE. Memory B cells become persistently re-activated in RA and SLE [65]. In memory B cells as well as in CD4^+ ^and CD8^+ ^memory T cells, *efhd2 *expression is part of an evolutionary conserved transcriptional signature [32]. Whether *efhd2 *expression in immune memory cells is important for their activation, to keep them silent, or to help them patrolling through secondary lymphatic organs is unknown at present but certainly deserves further investigation with respect to autoimmune diseases.

### EFhd2 and neuropathological disorders

Neurodegenerative disorders associated with tau, so-called tauopathies, are characterized by successive deposition of protein aggregates consisting of hyperphosphorylated tau [66,67]. In Parkinson's patients, tau and nitrated α-synuclein induce formation of so-called Lewy bodies that are characteristic for Parkinson's disease [68]. Tau aggregates and Lewy bodies cause synapse loss and neuronal cell death. In addition, neurodegenerative diseases reveal an inflammatory component represented mainly by microglial activation [69]. There are several lines of evidence that EFhd2 is involved in normal and pathological brain function: First, in microglia cells, EFhd2 becomes up-regulated and secreted in response to stimulation with nitrated α-synuclein [39]. Very interestingly, the related AIF-1 (Iba1) is a microglial activation marker [70]. Second, the brain of a FTDP-17 mouse model (JNPL3 mouse) [56] where neurodegeneration and neuroinflammation are induced through transgenic expression of a mutated human tau (htau P301L) contains complexes of the transgenic human tau protein and endogenous EFhd2 [15] (Figure 3). In addition, endogenous EFhd2 and tau associate in brain lysates of humans with Alzheimer's disease and FTDP-17 [15]. Third, EFhd2 is present in membrane rafts of mouse spinal cord, but only when mice over-express a mutant, toxic gain of function form of superoxide dismutase 1 (SOD1), namely the G93A mutant [71] (Figure 3). This mutant is responsible for familial amyotrophic lateral sclerosis, a chronic, progressive neuromuscular disorder [72]. Fourth, EFhd2 is down-regulated in Nogo-A knockout mice that reveal increased neurite growth and an increased regeneration potential when compared to wildtype control mice [73]. Fifth, in the frontal cortex of schizophrenic patients EFhd2 becomes up-regulated together with microtubule associated proteins [24]. Whether all these data represent a causative connection of EFhd2 with neurodegenerative diseases on the one hand, with neuronal regeneration on the other hand or are merely secondary effects is not known at present. Given the increasing incidence of neurodegenerative diseases, it would potentially be important to examine EFhd2 expression and function in human inflammatory diseases, including neuroinflammatory processes, and in murine mouse models for these diseases.

### Involvement of EFhd2 in membrane associated pathways

The plasma membrane contains cholesterol and sphingolipid-enriched microdomains (detergent resistant membranes, DRM) that are thought to function in many membrane-associated processes, such as immune receptor signaling [74]. In addition, microdomains control vesicular trafficking, signaling of various receptors other than immune receptors, and cytoskeletal rearrangement [75]. Microdomains and their associated scaffold proteins have also been implicated in the pathogenesis of several neurological diseases, such as Parkinson's and Alzheimer's disease [76] (see paragraph above). EFhd2 was discovered by us through a proteomic analysis of DRM of B cell lines that differ in their apoptotic response to BCR stimulation [30]. We found that EFhd2 associates with DRM in cell lines which undergo apoptosis after BCR stimulation [30] and indeed, as mentioned above, EFhd2 augments BCR signaling and BCR-induced apoptosis in one of these cell lines examined [14,31]. It was tempting to speculate that the DRM association of EFhd2 controls these effects. More recently we could actually show that the BCR-induced increase in intracellular calcium concentration is dependent on the DRM association of EFhd2 in WEHI231 cells [14]. However, EFhd2 was not associated with DRM in the murine B cell line CH27 [30]. We conclude that membrane raft association of EFhd2 is not constitutive but regulated, presumably in a cell type-specific manner. Hence, DRM association of EFhd2, and thereby its function, may be regulated by a specific pathway active in some cell types. There exist two lines of evidence for this hypothesis:

First, the predicted SH3-binding region (aa 70-78) of EFhd2, targeting EFhd2 to DRM, binds specifically to the SH3 domain of the tyrosine kinase Fgr [14] (Figure 3). In B cells, Fgr is only expressed late in B cell development [77] and there is only one study to our knowledge that examined DRM association of Fgr [78]. This study revealed that, in contrast to other Src family kinases, Fgr is not associated with DRM, at least in RAW264.7 cells [78]. Hence, once Fgr is expressed, it could keep EFhd2 out of DRM. The SH3 domain of Lyn that also binds to EFhd2 [14] could conversely maintain DRM association of EFhd2 (Figure 3). While Lyn is expressed throughout B cell development, Fgr is expressed in mature primary B cells, mantle zone B cells and during myelomonocytic differentiation, but not in immature primary B cells [77,79], which might regulate the subcellular localisation and the function of EFhd2 in mature B cells. Interestingly, we observed a phosphorylation-dependent interaction of EFhd2 with Lyn and Fgr [14]. Phosphorylation of murine and human EFhd2 at serine residues 74 and 76 [80-83] and, in brain lysates, of tyrosine 83 [38], have been described. Lipopolysaccharide stimulation of macrophages does not alter the phosphorylation of EFhd2 significantly [83]. In our hands, EFhd2 was not phosphorylated on tyrosine before or after BCR engagement as judged by western blotting with the anti-pY mAb PY99 (D.M. et al., unpublished data). In conclusion, phosphorylation of either serine 74 or 76, or both, or other sites would appear to mediate binding of EFhd2 to the SH3 domains of Fgr and Lyn. Interestingly, S74 and S76 of EFhd2 are Cdk1 (cyclin dependent kinase 1) phosphorylation sites [80]. Cdk1 is expressed and active during the G2/S phase of the cell cycle [84], that is, in activated B cells, which are present for instance in germinal centers [85]. Hence, EFhd2 could be modified by phosphorylation to modulate BCR signals in cycling B cells where the BCR induces G1 arrest and apoptosis [86], both of which are enhanced by EFhd2 [31].

Second, EFhd2 is present in DRM of mouse spinal cord, but only when mice over-express a mutant, toxic gain of function form of superoxide dismutase 1 (SOD1), namely the G93A mutant [71]. This mutant is responsible for familial amyotrophic lateral sclerosis, a chronic, progressive neuromuscular disorder [72]. Interactions of DRM with the cytoskeleton have been well documented [75]. Interestingly, the G93A mutant of SOD1 also induces DRM association of cytoskeletal proteins, such as ezrin, clathrin, actin depolymerising factor and Arp2/3 [71] (Figure 3). In accordance, EFhd2 has been identified in a complex with ezrin [55]. Under certain yet to be defined circumstances, EFhd2 may target ezrin, clathrin and others to DRM or become targeted to DRM by cytoskeletal proteins. De-phosphorylation of ezrin after BCR-triggering is important to induce DRM association of the BCR at late time points (more than 15 min) [87]. We have also shown that, at least in the murine B cell line WEHI231, EFhd2 induces constitutive association of the BCR, PLCγ2 and Syk with DRMs [14]. Taken together, EFhd2 may function as a DRM - cytoskeleton integrator through as yet not well characterized protein-protein or protein-lipid interactions.

### Proposed function(s) of EFhd2

As detailed above, EFhd2 is a cytoskeleton associated adaptor protein involved in immune and normal as well as pathological neuronal functions, and perhaps in calcium homeostasis. This may be important for immune cell signaling and signaling pathways within or arising from neurological synapses. In accordance, EFhd2 is regulated under inflammatory conditions in immune and brain cells. Hence, we propose that EFhd2 is a modulator of immune and brain cell function under both basal and inflammatory conditions.

## EFhd1

### Expression of EFhd1

EFhd1 was first described as mitocalcin. It was identified in an attempt to isolate genes involved in neuronal differentiation in 2Y-3t cells, a neuronal progenitor cell line established from an adult p53-deficient mouse cerebellum [88]. In 2Y-3t cells EFhd1 is up-regulated during differentiation. *In vivo*, EFhd1 is expressed both in cerebellum and cerebrum [16,88]. Its expression increases postnatally. In adult mice, neurons of cerebellum, cerebrum and hippocampus (cornu ammonis and dentate gyrus) reveal *efhd1 *mRNA, but only low amounts are present in the white matter of cerebellum and cerebrum [88]. The Purkinje-layer, the internal granule cell layer and the molecular layer, however, do express EFhd1. In addition, spermatocytes, interstitial cells of adult testis and granulosa cells of the cumulus oophorus (cumulus cells) as well as mural granulosa cells in adult ovary exhibit EFhd1 expression [16,88]. EFhd1 is also expressed in IGF-II-deficient murine C2 myoblasts [89]. There, *efhd1 *mRNA ceases in the absence of the transcriptional co-activator p300 [89]. Healthy human renal tissue expresses *efhd1 *mRNA [90] and in murine kidney the collecting ducts, but not the glomeruli, reveal EFhd1 [16,88]. Interestingly, EFhd1 expression is repressed in renal cell carcinoma (RCC) [90]. In contrast, EFhd1 is present in tumour tissues from patients with stage III and IV melanoma and increased expression of EFhd1 is significantly associated with shorter patient survival times [91]. Whereas EFhd1 has been proposed to exhibit tumour suppressing functions in RCC [90], growth promoting functions may be associated with other tumours. Concomitantly, uterine fibroids reveal less *efhd1 *mRNA than normal myometrium [92]. Under more physiological conditions EFhd1 becomes up-regulated at term when compared with midgestation [93]. In contrast, human endometrial cells down-regulate EFhd1 after trophoblast co-culture [94].

### Function of EFhd1 in normal and malignant tissue

**Figure 4:**
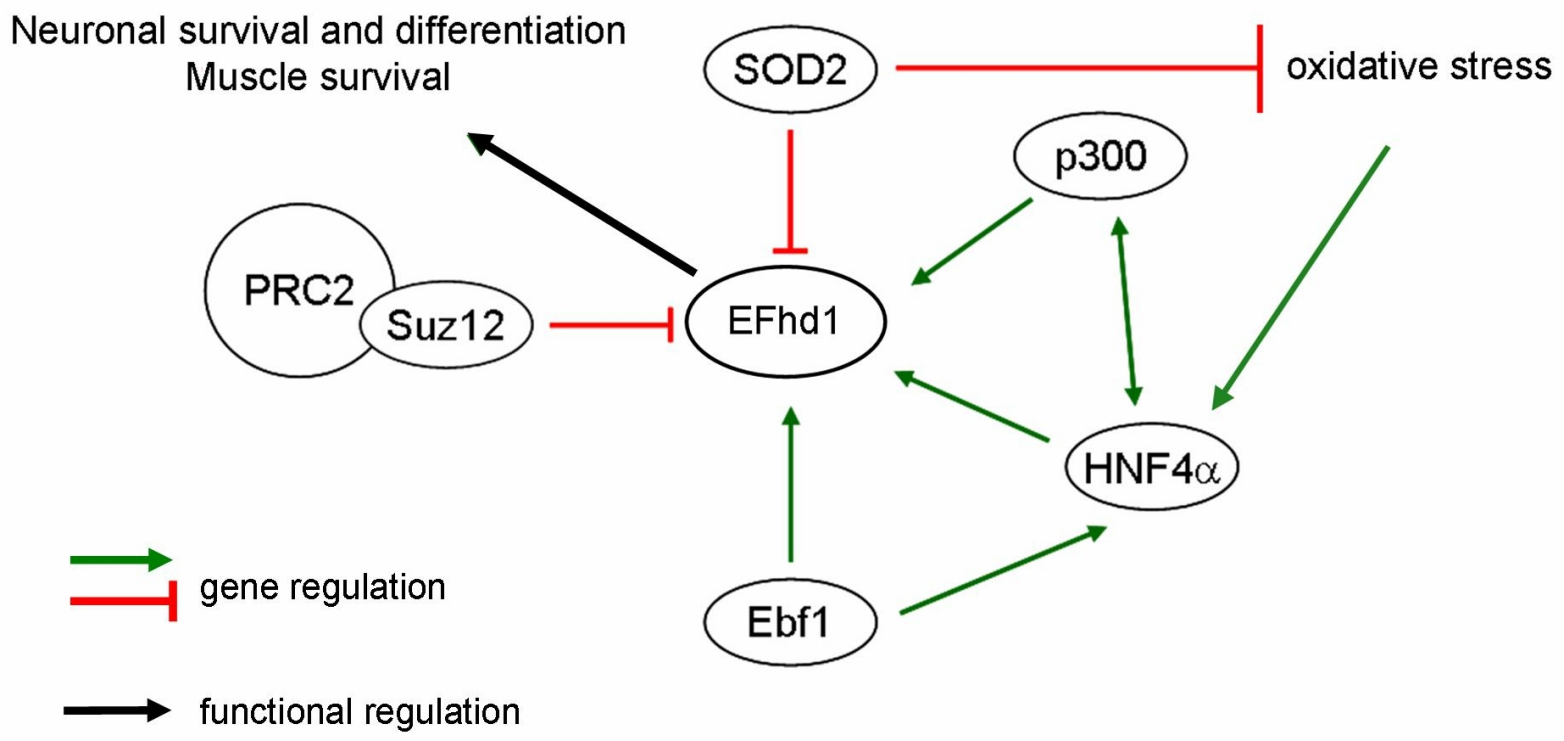
**Involvement of EFhd1 in survival and redox regulated pathways**. EFhd1 has been described as a survival factor for neuronal cells and been proposed to mediate muscle cell survival. In response to oxidative stress that can be blocked by superoxide dismutase 2 (SOD2), HNF-4α (hepatocyte nuclear factor 4α) becomes activated. EFhd1 is a direct target of HNF-4α and its expression is repressed by SOD2. Thus, EFhd1 may be involved in a protective cellular response against oxidative stress and, thereby, exhibit anti-apoptotic functions in diverse cell types. Early B cell factor 1 (Ebf1) positively regulates EFhd1 expression in early B cells whereas Suz12 as part of the polycomb repressor complex 2 (PRC2) is a negative regulator of EFhd1 expression.

Using an SDS-gel shift assay EFhd1 has been shown to bind calcium [88]. It associates with the mitochondrial inner membrane [16] and over-expression results in neurite extension of 2Y-3t cells. Conversely, down-regulation of EFhd1 through siRNA suppresses neurite outgrowth and promotes cell death [16]. Based on these findings Tominaga and co-workers suggested that EFhd1 is involved in neuronal differentiation and operates through control of mitochondrial function [16]. Whether this proposed function correlates with the calcium binding activity of EFhd1 is not known at present. Mitochondria, however, are important reservoirs of calcium. Interestingly, ectopic expression of manganese superoxide dismutase 2 (SOD2), a mitochondrial enzyme that inactivates superoxide to protect cells from oxidative damage, induces down-regulation of EFhd1 in a pancreatic carcinoma cell line. Conversely, reduction of SOD2 abundance causes increased EFhd1 expression in a human pancreatic carcinoma cell line (MIA-PaCa2) [95]. At first glance it is therefore puzzling that reduction of SOD2 increases neuronal cell death by activating cell death pathways [96,97]. An explanation could be that up-regulation of EFhd1 through reduced SOD2 expression [95] may be part of an anti-apoptotic mechanism antagonizing oxidative stress in neuronal cells (Figure 4). An example of a small calcium binding protein antagonizing oxidative stress is calmodulin: After calcium binding, calmodulin interacts with antioxidant enzymes involved in ROS homeostasis [1]. A similar mechanism may apply to EFhd1 in a tissue-specific manner.

Sustained activation of the autocrine IGF-II pathway is critical for myoblast viability and differentiation [98]. Gene expression profiling of IGF-II-deficient myoblasts that undergo apoptosis when incubated in differentiation-promoting medium revealed new mediators of CBP/p300 promoted survival. Myoblasts expressing CBP/p300 showed a 17-fold up-regulation of EFhd1 [89]. This indicates that EFhd1 might be a factor causing CBP/p300 mediated survival in muscle cells. Interestingly, together with PCAF, the transcriptional co-activator CBP/p300 is a key player in neuronal outgrowth by acetylating histones and p53 [99] and exhibits neuroprotective functions [100]. It is tempting to speculate that CBP/p300 up-regulates EFhd1 also in neuronal tissue to promote neuronal survival.

As readily mentioned above, EFhd1 expression has also been described in kidney. This is repressed in renal cell carcinoma (RCC) when compared to healthy tissue [90]. The same holds true for the transcription factor HNF4α (hepatocyte nuclear factor 4α). This suggests that HNF4α exhibits tumour suppressing functions [101]. Interestingly, HNF4α increases expression of EFhd1, revealing that *efhd1 *is a target gene of HNF4α [90,102]. Very recently, an independent microarray analysis confirmed this initial finding [103]. Ectopic HNF4α-expression reduces proliferation in HEK293 cells (a human embryonic kidney cell line) and therefore, the authors postulate a possible tumour suppressor activity of HNF4α [102]. However, inducible EFhd1 expression in HEK293 cells revealed that EFhd1 rather augments proliferation slightly [102]. Therefore, *efhd1 *is a HNF4α target gene that appears to oppose the proliferation suppressing effect of HNF4α in HEK293 cells. Additionally, HNF4α up-regulates hepatocyte iNOS (inducible nitric oxide synthetase) in response to the inflammatory redox state [104,105]. iNOS exhibits anti-oxidant and anti-apoptotic functions. Hence, it is likely that HNF4α senses the inflammatory redox state in tissues where it is expressed. Taken together, the mitochondrial localization of EFhd1, its ability to promote differentiation of 2Y-3t cells and its converse regulation by the redox factors SOD2 and HNF4α suggest that EFhd1 is part of a redox-sensitive network controlling cell survival and/or differentiation (Figure 4). Whether EFhd1 is actually a survival promoting or tumour suppressing factor may depend on the cell type and environmental context. For example, under pathological conditions, such as hypoxia and ischemia/reperfusion injury, mitochondrial dysfunction results in ROS increase. Normal tissue responds to this challenge with calcium overload, followed by mitochondrial depolarization, cytochrome c release and apoptosis [1]. Tumours, however, have developed mechanisms to survive hypoxia and ROS increase. One of these factors might be EFhd1.

A combined genome-wide ChIP sequencing analysis with gain- and loss-of-function transcriptome analyses in early-stage B cells identified promoters of genes with Ebf1 (early B cell factor-1) binding sites. IL-7 cultured pro-B cells, in which *Ebf1 *was conditionally inactivated, express less EFhd1 [106], suggesting that *efhd1 *is a target gene of Ebf1. *Hnf4α *was also described as direct target of the transcription factor Ebf1 in common lymphoid progenitors [107] and common lymphoid progenitors are subject to oxidative stress [108]. Thus, Ebf1 may up-regulate EFhd1 through HNF4α. These data point to a function of EFhd1 during early B cell development, an issue we are currently addressing. In contrast to transcriptional activators of *efhd1*, Suz12 is a transcriptional repressor as part of the transcriptional repressor complex PRC2 (polycomb repressor complex) in murine terato-carcinoma cells [109]. These complexes are normally highly abundant in embryonic tissues and are essential for development. In adult tissues, expression of Suz12 is very low [110,111] but it is very abundant in a variety of human tumours. PRC2 catalyzes trimethylation (me3) of histone 3 lysine 27 (H3K27), thereby, mediating transcriptional repression. Consequently, targeting of the *efhd1 *promoter by Suz12 should lead to decreased or no expression of EFhd1.

## Conclusions

Calcium binding proteins of the EF-hand are involved in all aspects of cellular functions. EFhd2 and EFhd1 are two related EF-hand containing adaptor proteins with a similar predicted overall structure. Both proteins bind calcium, but they differ in their sites of expression as well as in their proposed function in different cell types. EFhd2 is a cytoskeleton associated adaptor protein involved in immune and brain cell function and acts likely in response to changes in calcium homeostasis. EFhd1 is involved in neuronal differentiation and operates through control of mitochondrial function. Furthermore EFhd1 could be part of a redox-sensitive network controlling cell survival and/or differentiation depending on the cell type and environmental context. Whereas EFhd1 and EFhd2 are almost identical in the C-terminal part containing the EF-hands and the coiled-coil domain, a non-homologous moiety between aa 20-80 in front of the EF-hands shows the most differences between EFhd2 and EFhd1, as well as between orthologues of EFhd2 and EFhd1. As a result, EFhd2 and EFhd1 may bind distinct proteins redundantly while others uniquely, thereby, transmitting alterations in the intracellular calcium concentration into different signaling outcomes. Hence it appears comprehensible that expression of EFhd2 and EFhd1 is tightly controlled and regulated to ensure efficient function of the respective cell type. Besides the compelling necessity to clarify the detailed functions and signaling pathways of EFhd2 and EFhd1 in different cellular contexts, future work has to address possible redundancies of EFhd2 and EFhd1. Therefore, it is reasonable to generate and cross EFhd2 knockout and transgenic mice with the respective Efhd1 mice and analyze the impact in function and development, especially with regard to the immune and nervous system, as well as the progression of tumours.

## Abbreviations

aa: amino acid(s); AIF-1: Allograft inflammatory factor-1; BAFF: B cell activating factor of the TNF family; BCR and TCR: B and T cell receptors; Ca^2+^: Calcium ions; Cdk1: cyclin dependent kinase 1; CRAC: calcium release activated calcium channels; DRM: detergent resistant membranes; Ebf1: early B cell factor-1; EGF: epidermal growth factor; ER: endoplasmic reticulum; FTDP-17: frontotemporal dementia and parkinsonism linked to chromosome 17; GPCR: G-protein coupled receptors; HNF4α: hepatocyte nuclear factor 4α; Ig: immunoglobulin; ITAM: immunoreceptor tyrosine based activation motif; iNOS: inducible nitric oxide synthetase; IP_3_: inositol-1,4,5-trisphosphate; Iba1: ionized calcium-binding adapter molecule 1; LPS: lipopolysaccharide; NK cells: natural killer cells; PAMP: pathogen associated molecular patterns; PBMC: peripheral blood mononuclear cells; PMA: phorbol myristyl acetate; PIP_2_: phosphatidylinositol 4,5 bisphosphate; PRC2: polycomb repressor complex; ROS: reactive oxygen species; RANK-L: receptor activator of NF-κB ligand; RCC: renal cell carcinoma; RA: rheumatoid arthritis; SOCE: store operated calcium entry; STIM: stromal interaction molecule; SOD1/2: superoxide dismutase 1/2; EFhd2: Swiprosin-1/EF-hand containing 2; EFhd1: Swiprosin-2/EF-hand containing 1; UTR: untranslated region

## Competing interests

The authors declare that they have no competing interests.

## Authors' contributions

SD and DM collected data, carried out sequence alignments, drafted figures and wrote the manuscript. SB collected data and wrote the manuscript. DM designed and coordinated the study. All authors read and approved the final manuscript.

## Supplementary Material

Additional file 1 **Table S1 Murine EFhd1 and EFhd2 genes, transcripts and proteins**. This table displays the nomenclature of murine EFhd1 and EFhd2 proteins and transcripts as well as gene locus information. Click here for file

Additional file 2 **Figure S1 Aligment of EFhd1 Orthologues**. Aligment of human (Q9BUP0), murine (Q9D41J), rat (D4A9T5), bovine (Q17QM6) and frog (Q6GP23) EFhd1 using ClustalW2 (http://www.ebi.ac.uk/clustalw). Amino acid (aa) positions are marked on the right. "*", identical aa, ":", conserved aa, "." semi conserved aa. Click here for file

Additional file 3 **Figure S2 Sequence homology of murine AIF-1 with EFhd1 and EFhd2**. A standard blast search with murine AIF-1 (AAC82481.1) was performed against all murine non-redundant GenBank CDS translations + PDB + SwissProt + PIR + PRF excluding environmental samples from WGS projects. Homologies between the first EF-hand of AIF-1 and the EF-hands of EFhd1/2 are only shown for the second EF-hands of EFhd1 and EFhd2. Click here for file
