# Supplementary material for: Fraternal twins: Swiprosin-1/EFhd2 and Swiprosin-2/EFhd1, two homologous EF-hand containing calcium binding adaptor proteins with distinct functions
Source: Cell Commun Signal. 2011 Jan 18;9:2. doi: 10.1186/1478-811X-9-2 (PMC3036668; doi:10.1186/1478-811X-9-2)
Supplement: Additional file 1 — Table S1 Murine EFhd1 and EFhd2 genes, transcripts and proteins. This table displays the nomenclature of murine EFhd1 and EFhd2 proteins and transcripts as well as gene locus information. [file 1478-811X-9-2-S1.DOC]

| **Protein name and size** | **Synonyms for genes and proteins** | **Gene name** | **Gene Locus (Mus musculus)** | **Coding transcript ID and length** |
| --- | --- | --- | --- | --- |
| EFhd2, 240aa | 2600015J22Rik, AA408606, D4Wsu27e, EF-hand domain-containing protein D2, swiprosin 1, Swiprosin-1, Sws1, [ENSMUSP00000044502](http://www.ensembl.org/Mus_musculus/Transcript/ProteinSummary?db=core;g=ENSMUSG00000040659;r=4:141414057-141430835;t=ENSMUST00000036854) | *efhd2* | [Chromosome 4: 141,414,057-141,430,835](http://www.ensembl.org/Mus_musculus/Location/View?db=core;g=ENSMUSG00000040659;r=4:141414057-141430835;t=ENSMUST00000036854) reverse strand | [ENSMUST00000036854](http://www.ensembl.org/Mus_musculus/Transcript/Summary?db=core;g=ENSMUSG00000040659;r=4:141414057-141430835;t=ENSMUST00000036854), 2381b |
| EFhd1, 240aa | 4931430I01Rik, AI452351, EF-hand domain-containing protein 1, EF-hand domain-containing protein D1, MGC103094, mitocalcin, PP3051, Swiprosin-2, Sws2 | *efhd1* | [Chromosome 1: 89,160,938-89,207,414](http://www.ensembl.org/Mus_musculus/Location/View?db=core;g=ENSMUSG00000026255;r=1:89160938-89207414) forward strand | [ENSMUST00000027472](http://www.ensembl.org/Mus_musculus/Transcript/Summary?db=core;g=ENSMUSG00000026255;r=1:89160938-89207414;t=ENSMUST00000027472), 1864b  [ENSMUST00000118687](http://www.ensembl.org/Mus_musculus/Transcript/Summary?db=core;g=ENSMUSG00000026255;r=1:89160938-89207414;t=ENSMUST00000118687), 1726b  [ENSMUST00000150831](http://www.ensembl.org/Mus_musculus/Transcript/Summary?db=core;g=ENSMUSG00000026255;r=1:89160938-89207414;t=ENSMUST00000150831) |
