# Supplementary figures and images for: Fraternal twins: Swiprosin-1/EFhd2 and Swiprosin-2/EFhd1, two homologous EF-hand containing calcium binding adaptor proteins with distinct functions
Source: Cell Commun Signal. 2011 Jan 18;9:2. doi: 10.1186/1478-811X-9-2 (PMC3036668; doi:10.1186/1478-811X-9-2)

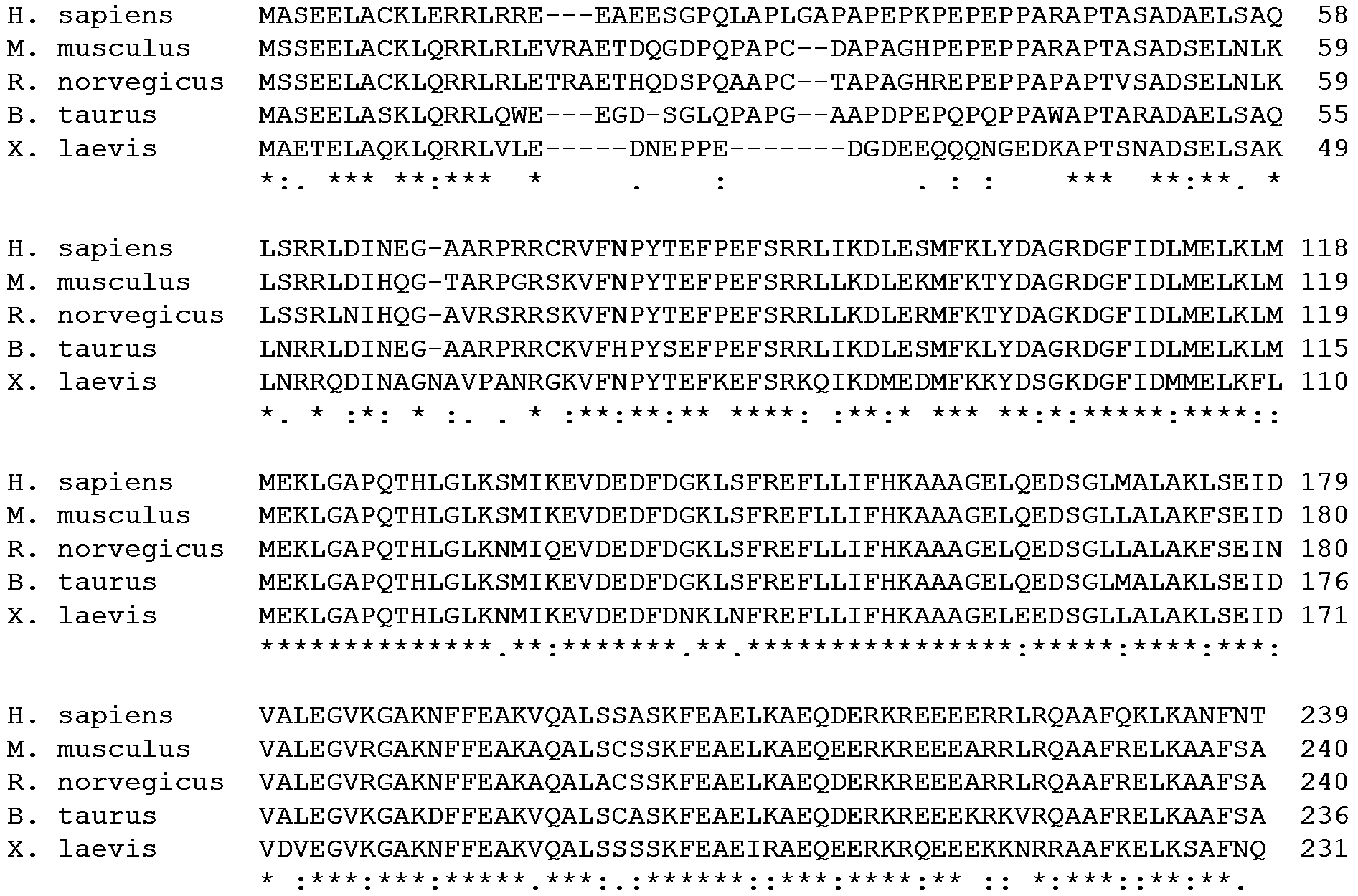

Supplement: Additional file 2 — Figure S1 Aligment of EFhd1 Orthologues. Aligment of human (Q9BUP0), murine (Q9D41J), rat (D4A9T5), bovine (Q17QM6) and frog (Q6GP23) EFhd1 using ClustalW2 (http://www.ebi.ac.uk/clustalw). Amino acid (aa) positions are marked on the right. "*", identical aa, ":", conserved aa, "." semi conserved aa. [file 1478-811X-9-2-S2.JPEG]

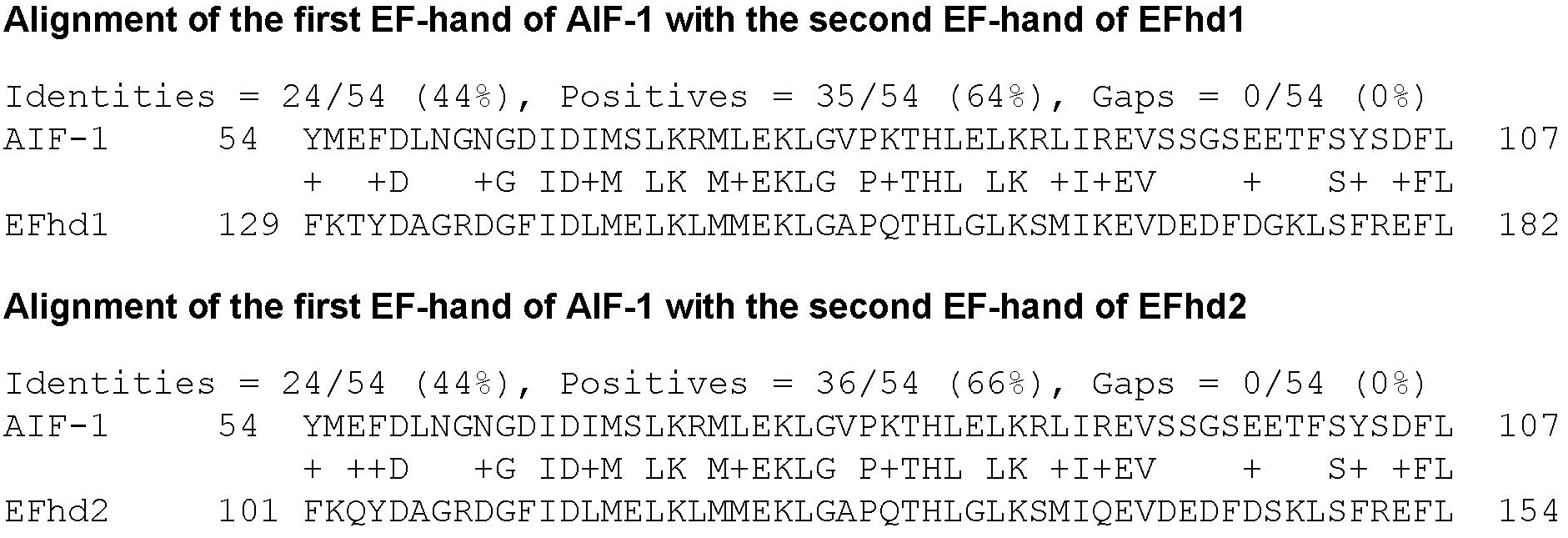

Supplement: Additional file 3 — Figure S2 Sequence homology of murine AIF-1 with EFhd1 and EFhd2. A standard blast search with murine AIF-1 (AAC82481.1) was performed against all murine non-redundant GenBank CDS translations + PDB + SwissProt + PIR + PRF excluding environmental samples from WGS projects. Homologies between the first EF-hand of AIF-1 and the EF-hands of EFhd1/2 are only shown for the second EF-hands of EFhd1 and EFhd2. [file 1478-811X-9-2-S3.JPEG]
